# Proteomic changes in oocytes after *in vitro* maturation in lipotoxic conditions are different from those in cumulus cells

**DOI:** 10.1038/s41598-019-40122-7

**Published:** 2019-03-06

**Authors:** Waleed F. A. Marei, Geert Van Raemdonck, Geert Baggerman, Peter E. J. Bols, Jo L. M. R. Leroy

**Affiliations:** 1 Gamete Research Centre, Department of Veterinary Sciences, University of Antwerp, 2610 Wilrijk, Belgium; 2 Department of Theriogenology, Faculty of Veterinary Medicine, Cairo University, Giza, 12211 Egypt; 3 Centre for Proteomics (CFP), University of Antwerp, Groenenborgerlaan 171, 2020 Antwerpen, Belgium; 4 VITO, Boeretang 200, 2400 Mol, Belgium

## Abstract

Maternal lipolytic metabolic disorders result in a lipotoxic microenvironment in the ovarian follicular fluid (FF) which deteriorates oocyte quality. Although cellular stress response mechanisms are well defined in somatic cells, they remain largely unexplored in oocytes, which have distinct organelle structure and nuclear transcription patterns. Here we used shotgun proteomic analyses to study cellular responses of bovine oocytes and cumulus cells (CCs) after *in vitro* maturation under lipotoxic conditions; in the presence of pathophysiological palmitic acid (PA) concentration as a model. Differentially regulated proteins (DRPs) were mainly localized in the endoplasmic reticulum, mitochondria and nuclei of CCs and oocytes, however the DRPs and their direction of change were cell-type specific. Proteomic changes in PA-exposed CCs were predominantly pro-apoptotic unfolded protein responses (UPRs), mitochondrial and metabolic dysfunctions, and apoptotic pathways. This was also functionally confirmed. Interestingly, although the oocytes were enclosed by CCs during PA exposure, elevated cellular stress levels were also evident. However, pro-survival UPRs, redox regulatory and compensatory metabolic mechanisms were prominent despite evidence of mitochondrial dysfunction, oxidative stress, and reduced subsequent embryo development. The data provides a unique insight that enriches the understanding of the cellular stress responses in metabolically-compromised oocytes and forms a fundamental base to identify new targets for fertility treatments as discussed within.

## Introduction

Maternal metabolic disorders such as obesity and type-II diabetes are increasing in prevalence and have been strongly linked with reduced fertility^1^ and IVF success rates^2,3^. Trials using metabolically-healthy donor oocytes could improve the fertility of those patients^4,5^. Thus, direct effects on oocyte quality is considered an important link between maternal metabolic diseases and reduced fertility. Understanding the underlying mechanisms that occur at the oocyte level is important to increase treatment efficiency.

The ovarian follicular fluid (FF) composition is significantly altered by metabolic factors^2,6^. Upregulated lipolysis is common in metabolic disorders e.g. obesity and type-II diabetes due to reduced insulin sensitivity. Elevated non-esterified free fatty acid (FFA) concentrations in blood, predominantly; palmitic (PA), stearic, linoleic and oleic acids are reflected in the FF^2,7^. Particularly, high levels of PA in FF was linked with negative pregnancy outcome following ICSI^8^. PA-induced lipotoxicity causes various pathological conditions e.g. cardiovascular and neurological diseases^9^. We have previously shown, using mouse and bovine models, that *in vitro* exposure to pathophysiological concentrations of FFAs compromise oocyte quality and significantly reduce oocyte developmental competence^10,11^, leading to lower proportions of transferable embryos, higher apoptotic cell indices^12^, and significant alterations in embryo epigenetic and transcriptomic profiles^13^.

Cumulus cells have been shown to accumulate cytoplasmic lipid droplets from their microenvironment^14^. Intracellular lipid accumulation in somatic cells causes abnormal cellular metabolism, lipid peroxidation, leading to oxidative stress and protein misfolding^15^. Accumulation of misfolded proteins in the endoplasmic reticulum (ER) causes ER stress that elicit specific “unfolded protein response” (UPRer)^16^. This is evident in FFA-treated bovine COCs *in vitro*^17^ particularly in CCs^12^ and was associated with reduced embryo development rates. Similar responses occur in the mitochondria (UPRmt)^18^. These UPRs stimulate nuclear expression of chaperons to maintain cellular survival or induce programmed cell death if stress level is intolerable^19^. Although our understanding of these mechanisms is expanding in somatic cells, it remains largely unexplored in oocytes, which have different organelle composition and nuclear transcription compared to somatic cells^20^. Studying these regulatory mechanisms in details in oocytes involves complex networks and can be challenging considering the scarceness of samples and ethical limitations in humans. Bovine oocytes are available in large numbers from slaughter house material and share many functional similarities with human oocytes e.g. the duration of folliculogenesis, oocyte diameter, lipid content, timing of maturation, early cleavage, and embryo genome activation^21^, and thus is proposed to be a good model for studying molecular responses to metabolic stress.

We have previously observed differential expression of few genes related to lipid and carbohydrate metabolism between CCs and oocytes derived from FFA-treated bovine COCs^22^. However, considering that most cellular stress responses involve changes at the protein level^23^, transcriptomic analysis may not be comprehensive. Recent advancement of proteomic analysis has increased the sensitivity and accuracy of identification and quantification of proteins in small sample size. Therefore, the aim of this study was to analyze and compare cellular responses to PA-induced lipotoxicity during *in vitro* maturation of bovine COCs using shotgun proteomic analysis of CCs and of the enclosed-oocytes. This was complemented by other functional tests of mitochondrial activity, oxidative stress levels, cellular apoptosis and a follow up of subsequent early embryo development.

## Results

### Effect of PA-exposure during IVM on oocyte developmental competence and embryo quality

Good quality bovine COCs were isolated and selected from ovaries of slaughtered cows and matured *in vitro* in the presence of a pathophysiological concentration of PA (150 µM) or solvent (ethanol, 0.01%). PA significantly (*P* < 0.05) reduced total cleavage rates and proportions of good-quality embryos (4-cell-stage or beyond) at 48 h post-insemination (p.i.), while embryo fragmentation was increased compared to the solvent controls (*P* < 0.05). At day 8, blastocyst rates were significantly lower in the PA group compared to the solvent control (*P* < 0.05) despite fertilization and culture in a FA-free condition (Table 1). We have previously shown that ethanol (0.01%) does not affect oocyte quality or embryo development if compared to solvent-free control^12^. PA-derived blastocysts were lower in quality as they exhibited higher percentage of apoptotic cells (CASP3 positive), mainly in the inner cell mass (ICM), compared to solvent control. The average counts of total, ICM, and trophectoderm (TE) cells were not affected by the treatment as determined by differential immunostaining with CDX2 and Hoechst.

**Table 1:** *In vitro* early development and quality of embryos derived from bovine COCs exposed to PA (150 µM) or solvent (ethanol, 0.01%) during IVM.

	Solvent	PA	*P* value
Total (from 4 replicates)	295	308	
Total Cleaved embryos, n(%)	230 (77.9%)	196 (63.6%)	<0.001
Good quality embryos (≥4-cells), n(%)	171 (57.9%)	115 (37.3%)	<0.001
Fragmented embryos, n(%)	13 (4.4%)	32 (10.3%)	0.005
D8 total blastocysts, n(%)	94 (31.8%)	65 (21.1%)	0.003
Total cell counts*	128 ± 8	116 ± 11	0.393
Trophectoderm (TE)*	87 ± 5	82 ± 8	0.620
Inner cell mass (ICM)*	42 ± 4	35 ± 3	0.233
Total Apoptosis^□^	4.2 ± 0.78	12.7 ± 2.84	0.004
Apoptosis in TE^□^	3.6 ± 0.75	7.7 ± 2.68	0.309
Apoptosis in ICM^□^	5.9 ± 1.7	25.6 ± 5.14	<0.001

*Cell counts are shown as mean ± SEM, while ^□^ apoptosis is shown as the percentage ± SEM of CASP3 positive cells from total cell counts.

### Effect of PA on apoptosis in COCs

**Figure 1:**
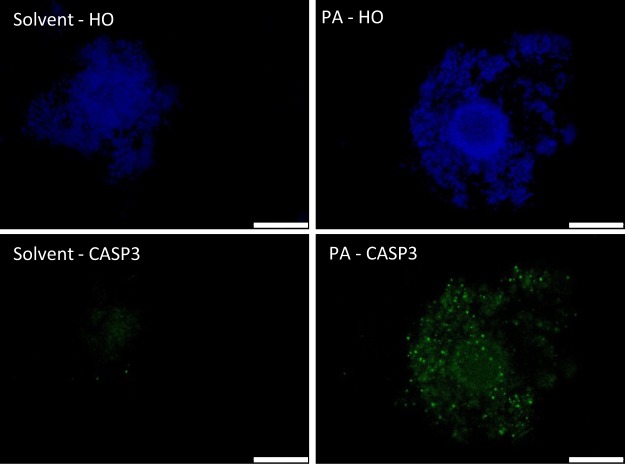
Immunocytochemical staining of cleaved-caspase-3 (CASP3) expression in COCs matured *in vitro* in the presence of PA (150 µM) or solvent (ethanol, 0.01%). Nuclei are counterstained with Hoechst (HO). Scale bar = 200 µm.

Apoptosis was higher (P < 0.05) in CCs in the PA-exposed COCs compared to those in solvent control. 11/34 (32.3%) of PA-COCs had ≥25–50% of their CCs positively stained to CASP3, and 17/34 (50%) of the COCs had 10–25% of the cells apoptotic. Whereas, the majority of COCs in solvent control (34/35, 97.1%) showed very low CASP3 staining (0–5%). No CASP3 positive staining could be detected in the oocytes in both treatment groups (Fig. 1).

### Effect of PA on mitochondrial activity and oxidative stress in COCs

**Figure 2:**
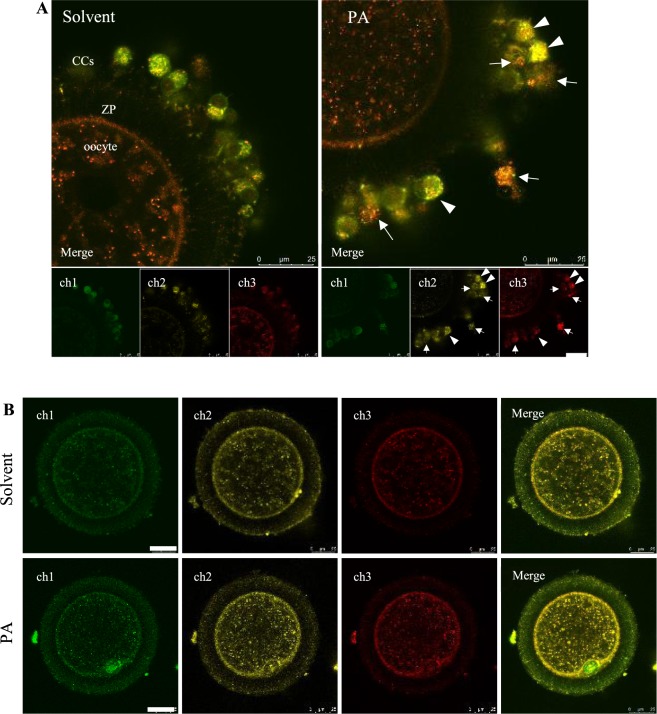
Combined JC-1 and CellRox^TM^ deep red staining of CCs (2A) and oocytes (2B) from PA and solvent control COCs. ch1, JC-1 monomers (525 nm); ch2, JC-1 aggregates (590 nm); ch3, CellRox staining. In (**A**), arrows indicate cells with high CellRox and low J-aggregate intensity, and arrow heads indicate cells with high CellRox and high J-aggregates intensity. CCs, cumulus cells; ZP, zona pellucida. Scale bar = 25 µm.

CellRox^TM^ deep red staining intensity (as an estimate for intracellular reactive oxygen species, ROS) was significantly higher in PA-treated CCs (29.7 ± 3.87 vs. 17.5 ± 0.74, *P* < 0.05) compared with the solvent controls. This staining was done in combination with JC1 to determine mitochondrial activity. J-aggregates: monomers (590: 525 nm) ratio (as an estimate of inner mitochondrial membrane potential, MMP) was also significantly higher in PA-treated CCs (108.5 ± 4.10% vs. 74.7 ± 4.71% in solvent control). However, we noticed that JC-1 and CellRox staining intensity in PA-treated CCs were markedly heterogeneous within the same COC while more homogenous in control COCs (Fig. 2A). Interestingly, some cells exhibiting high oxidative stress level (CellRox staining, red) had the lowest J-aggregate fluorescence intensity (yellow) suggesting loss of MMP, i.e. uncoupling (see cells indicated with arrows, Fig. 2B). Other CCs exhibiting high MMP had high CellRox staining (arrow heads, Fig. 2A).

In oocytes, MMP was significantly higher in the PA group compared to controls; where the average J-aggregates: monomer ratio was 144 ± 4.1 vs. 123 ± 5.1 (*P* < 0.05). CellRox intensity was also higher in PA-oocytes (43.3 ± 2.36 vs. 28.4 ± 2.43, *P* < 0.05) indicating higher levels of oxidative stress (Fig. 2B). An obvious co-localization was noticed between CellRox and JC-1 staining in the oocytes in both treatment groups (see merged images).

### TMT proteomic analysis: Differentially regulated proteins in response to PA-treatment

**Figure 3:**
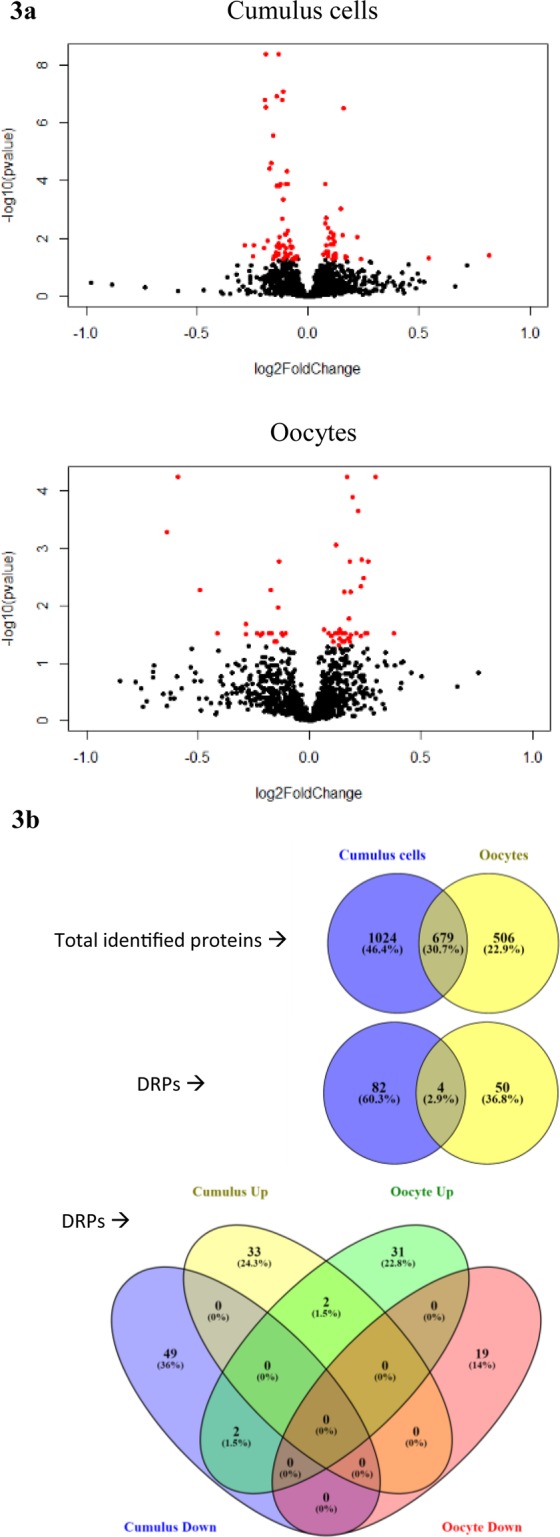
Global analysis of proteomic responses to PA-treatment in CCs and oocytes. (**a**), Volcano plots showing log fold changes vs. −log10 p values for the effect of PA-exposure on the relative abundance of the identified proteins in CCs and oocytes. Differentially regulated proteins (DRPs; PA vs. Solvent) with adjusted *P* value < 0.05 and fold change >10% are depicted in red. (**b**), Venn diagrams show the number of total number of identified proteins in both treatment groups in CCs and oocytes, and the number of PA-induced DRPs (UP or DOWN regulated compared with Solvent) in each cell type.

A total of 1703 and 1185 proteins were identified in CCs and oocytes, respectively, 679 of which were common. *P* values and fold changes of these proteins are depicted in the volcano plots in Fig. 3. Exposure to PA resulted in a significant change in the relative abundance of 86 and 54 proteins (DRPs) in CCs and oocytes, respectively, compared to the solvent group. Only 4 DRPs were common in CCs and oocytes, which shows distinct cell type-dependent responses. Hierarchical clustering of the relative abundance of DRPs using MeV software showed close clustering of the three PA samples apart from the three control samples within the oocyte and CC databases.

### Cellular localization of differentially regulated proteins

The majority of the DRPs of CCs and oocytes were localized in ER, mitochondria and nuclei. Most of the DRPs in CCs were down-regulated in the PA group compared with solvent, while the majority of the DRPs in oocytes were upregulated. (Table 2).

**Table 2:** Cellular localization of differentially regulated proteins (up- and down- regulated) in CCs and oocytes.

Differentially regulated proteins	Cumulus cells			Oocytes		
	Total	Up	Down	Total	Up	Down
**Total in different cellular compartments**	**86**	**35**	**51**	**54**	**35**	**19**
Endoplasmic reticulum (GO:0005783)	24	3	21	11	6	5
-ER membrane network	0	0	0	2	0	2
Mitochondrion (GO:0005739)	20	4	16	12	8	4
-Mitochondrial inner membrane	9	1	8	4	4	0
-Mitochondrial outer membrane	2	0	2	0	0	0
Plasma membrane	5	2	3	4	1	3
Golgi apparatus (GO:0005794)	4	2	2	6	5	1
Cytoplasmic membrane-bounded vesicle (GO:0016023)	1	1	0	4	3	1
Nucleus (GO:0005634)	20	9	11	13	10	3
Endosome (GO:0005768)	3	1	2	2	2	0
Lysosome (GO:0005764)	1	0	1	5	2	3
Cytoskeleton (GO:0005856)	8	5	3	2	1	1
Spindle	0	0	0	2	1	1
Lipid droplet	1	1	0	1	1	0
Macromolecular complex (including ribonucleoproteins) (GO:0032991)	**7**	**7**	**0**	**5**	**2**	**3**

### Biological functions and Canonical pathway analysis

**Figure 4:**
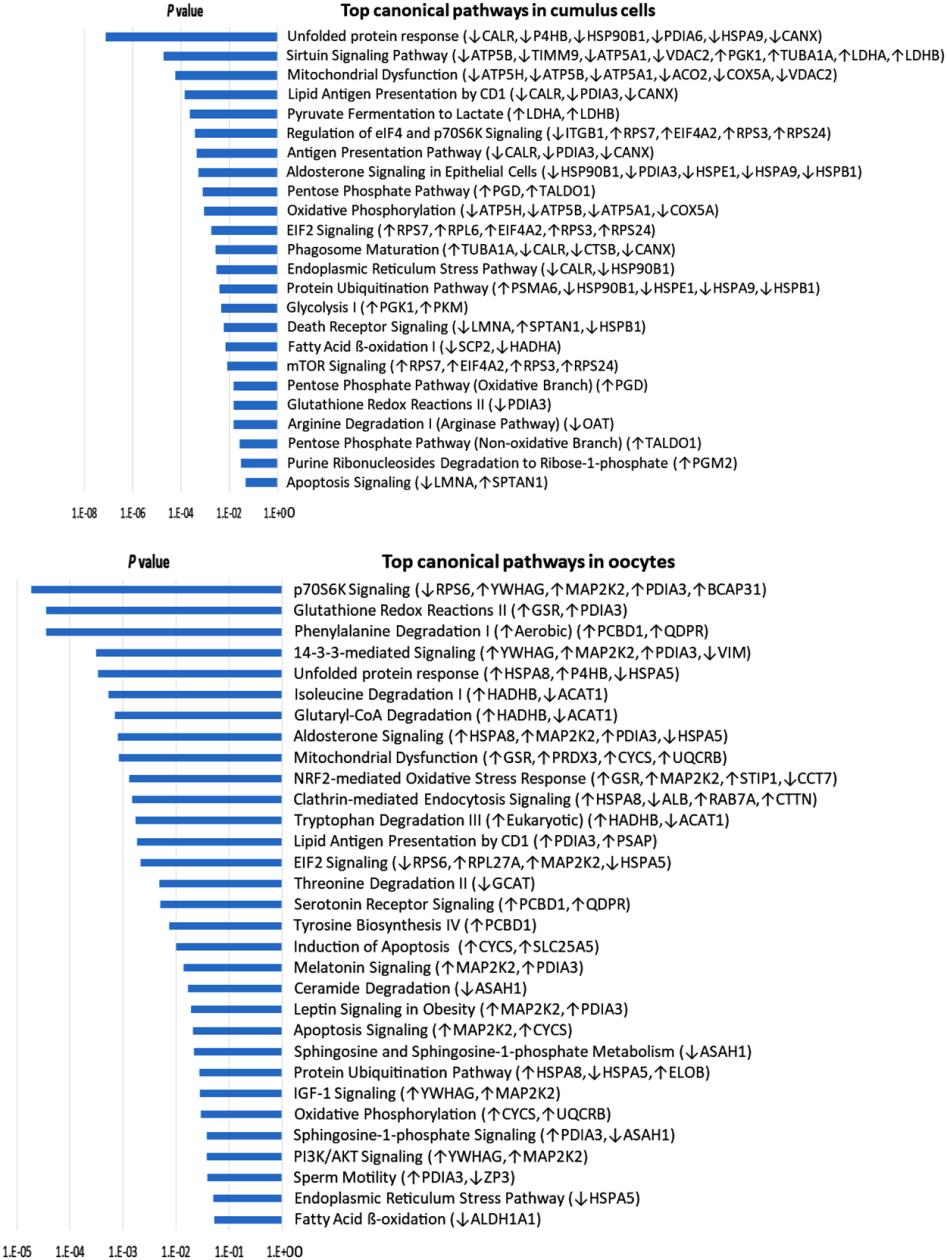
Top canonical pathways (with *P* < 0.05, generated by IPA) in PA-treated CCs and oocytes, and the proteins involved in each. “↑”, up-regulated; “↓”, down-regulated. See Supplementary Table 1a–d for full names, *P* values, fold changes and biological functions of each protein.

Core analysis in IPA (Qiagen Bioinformatics) showed that the PA-induced DRPs of CCs are involved in several canonical pathways related to cellular stress and metabolism. The top canonical pathways in CCs and the proteins involved in each pathway are listed in Fig. 4a. Interestingly, UPR, Sirtuin Signalling Pathway and Mitochondrial dysfunction pathways were the most affected. Other related pathways were also significantly induced by PA in CCs and include protein ubiquitination, EIF2 and mTOR signaling which are in line with the UPRs, as well as the influence on proteins involved in glycolysis, FA β-oxidation, and oxidative phosphorylation which altogether indicates a dysfunction in cellular metabolism.

In oocytes, UPR and mitochondrial dysfunction were also among the top pathways affected by PA treatment. However, interestingly, other specific pathways were prominent (Fig. 4b). The top significant pathway was the p70S6K signaling. Other affected pathways related to oxidative stress regulation were also significant e.g. glutathione redox regulation and NRF2-mediated oxidative stress response. To avoid repetition, these pathways are shown in Fig. 4 and described in the discussion. Lists of up and down regulated proteins in CCs and oocytes, and their individual biological functions, fold changes and *P* values are shown in details in Supplementary Tables 1a–d in Appendix 2.

**Figure 5:**
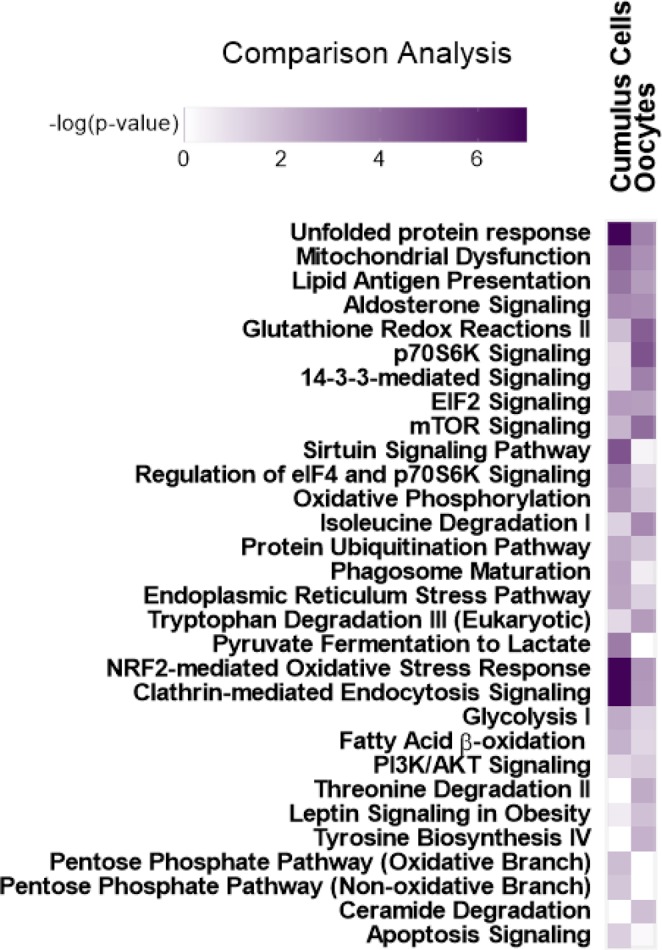
Comparison analysis between the top altered pathways in CCs and in oocytes due to PA-treatment during *in vitro* maturation. The comparison is based on the *P* value of each pathway generated by IPA software (Qiagen).

A comparison analysis of PA-induced DRPs in CCs vs. oocytes was also done in IPA (Qiagen Bioinformatics) (Fig. 5). Although the detected individual DRPs are different, many altered pathways were common. Interestingly, UPRs and mitochondrial dysfunction, as well as several metabolic pathways were among those common pathways. However, as shown in Fig. 4 and described in the discussion, the direction of change was opposite, particularly in UPR and ER stress pathways. Some pathways were exclusively or highly altered in CCs compared with the oocytes such as the Sirtuin signaling, pentose phosphate pathway (PPP), oxidative phosphorylation, and fatty acid β-oxidation. Whereas, other pathways were exclusively or highly altered in the oocytes such as p70S6K and 14-3-3-mediated signaling, and NRF-2 mediated oxidative stress response.

## Discussion

In the present study, we used TMT-labeled shotgun proteomics to gain a comprehensive understanding of the molecular stress response mechanisms in oocytes and CCs to PA-induced lipotoxicity. This experimental setup was used as a model for the lipotoxic follicular microenvironment in patients with lipolytic disorders where predominantly PA is elevated^2,8^. *In vitro* models provide limited representation of the follicular fluid biochemical alterations that occur *in vivo*, where interactions between different factors such as FFAs and other nutrients are evident and described^2,24–28^. However, strategically designed *in vitro* models enable focus on specific factors, pathways or mechanisms. The present study is a first trial to understand the stress response mechanisms to a lipotoxic insult in oocytes using a novel proteomic insight. We only focused on PA-induced lipotoxic effects to reduce the number of fixed factors, increase statistical power and allow accurate interpretation of the complex proteomic data in combination with other functional assessments. Interactions with other FFAs and hyperglycemic conditions during IVM have been described in our previous studies^11,12,28^.

Bearing in mind the complexity of the proteomic data, we focus in this discussion on top pathways and networks of biological relevance, and make speculations based on the functional annotations of the involved proteins and on the associated functional phenotypes. Detailed lists of the DRPs and their specific biological functions are supplemented for deeper insight.

First focusing on the responses in CCs, PA induced several alterations in cellular metabolism-related proteins, the majority of which were mitochondrial. Interestingly, three complementary subunits of the mitochondrial ATP synthase enzyme were simultaneously downregulated; ↓ATP5H is subunit d of the mitochondrial inner membrane-spanning component of ATP synthase complex; and ↓ATP5A1 and ↓ATP5B, which are subunits α1 and β of the soluble catalytic core of the enzyme complex. Mitochondrial Cytochrome c oxidase (Complex IV) subunit 5A (COX5A) was also downregulated. The reduced availability of complex IV and V subunits may have negative effects on the efficiency of the electron transport chain and ATP synthesis during oxidative phosphorylation in CCs. In addition, both lactate dehydrogenase A and B (LDHA and LDHB) were upregulated together with ↑PGK1 and ↑PKM suggesting higher rates of glycolysis and lactate production. We have previously shown that bovine COCs matured in high concentrations of palmitic, stearic and oleic acids (High NEFAs) had significantly higher *LDHA* mRNA expression in CCs^22^, and that bovine COCs matured under diabetes-like conditions (high glucose and NEFAs) produced more lactate during culture^29^. This is a mechanism known as the Warburg effect, which is common in cancer cells and has been described in non-cancerous conditions in association with tissue damage such as in meningitis, encephalitis, and acute pancreatitis^30^. Metabolism of glucose through the pentose phosphate pathway (PPP) also appears to be upregulated here in PA-treated CCs as suggested by ↑PGD, a member of the oxidative branch of PPP that generates NADPH, which in turn reduces glutathione to prevent oxidative stress. The NADPH-dependent enzyme, AKR1B1, that catalyzes reduction of aldehydes and ketones was also upregulated. ↑AKR1B1 has been linked to the pathogenesis of secondary diabetic complications such as neuropathy and nephropathy^31^. In the non-oxidative phase of PPP, ↑transaldolase 1 was observed, which is needed to generate pentoses for nucleotide synthesis.

In addition to these modifications in carbohydrate metabolism, FA β-oxidation (FAO) is also affected as suggested by ↓SCP2, a mitochondrial non-specific lipid transfer protein, and ↓HADHA, an inner membrane (IMM) mitochondrial trifunctional protein that is physically associated with complex I^32^. In addition, ↑FAF2, a lipase inhibitor, may inhibit triacylglycerol hydrolysis in PA-treated CCs. This may explain the increased lipid accumulation in NEFA-treated COCs^24^. Perhaps this reduced metabolic activity in the affected CCs is a cellular response to control oxidative stress, which is evident here as shown by the increased CellRox staining intensity in the PA group. Oxidative stress has been shown to reduce the relative abundance of complex I proteins in insulin-resistant skeletal cells that are involved in FAO^33^.

Besides the alterations in metabolism-related mitochondrial proteins, subunits of the “mitochondrial contact site and cristae organizing system” (MICOS): CHCHD3 and IMMT, were significantly reduced. This system is crucial for the formation and maintenance of cristae structure, and is involved in the formation of contact sites to the outer mitochondrial membrane (OMM) for fusion processes^34^. This may lead to deteriorated mitochondrial ultrastructure as reported in COCs of high fat diet-induced obese mice^35^.

As an expected consequence of oxidative stress, several ER and ribosomal proteins were dysregulated in CCs that are related to regulation of translation, protein transport, translocation and post-translational protein modification, as well as ER-to-golgi vesicle-mediated transport. This confirms the involvement of ER stress in the pathogenesis of lipotoxicity in CCs as previously suggested by Sutton-McDowall, *et al*.^17^. However, only few of these changes were pro-survival, while many pro-apoptotic changes were detected. Pro-survival changes include ↑FAF2 needed for ubiquitin-dependent ER-associated protein degradation “ERAD”; ↑RPS3 that is involved in HSP70 and HSP90 mediated regulation of translation; and ↓ERO1A^36^. EIF2α signaling was also upregulated, which is a rapid response to arrest protein translation^37^. In contrast, crucial pro-survival chaperons and proteins related to the UPR^er^ were downregulated in PA-exposed CCs, namely CALR, P4HB, HSP90B1, PDIA6, HSPA9, and CANX. This strongly suggests that ER-stress-induced cell death mechanisms are actively involved. High levels of ER stress can induce a process known as “regulated IRE1α-dependent decay” which degrades mRNA encoding for crucial proteins such as ER chaperons^38,39^. Programmed cell death in PA-treated CCs could also be facilitated by ↓VDAC2 (mitochondrial), ↓LRPAP1 (ER) and, ↓LMNA (nuclear), which are negative regulators of apoptosis. Previous reports have shown that PA induces apoptosis in granulosa cells^40^. Similarly, high percentage of CCs in the PA-treated COCs were apoptotic (positive to CASP3), but not all, which illustrates that different levels of ER stress are evident in PA-CCs. A heterogeneous pattern of mitochondrial activity and oxidative stress in different CCs within the same COC were also observed using JC1 and CellRox staining. These data suggest that preventative treatments aiming to reduce oxidative stress levels, inhibit ER stress signaling or enhance mitochondrial functions in CCs may protect oocyte developmental competence.

Now focusing on the oocytes, although they were enclosed (and protected^41^) by CCs during exposure to PA during IVM, significant alteration in the proteomic profile, oxidative stress levels and mitochondrial activity under PA-conditions *in vitro* clearly shows that the oocytes are also sensitive and responsive to the lipotoxic maturation conditions. The majority of PA-oocytes were cleaved after fertilization suggesting that the lipotoxic effect was less sever on oocytes compared to CCs. Importantly, although top canonical dysregulated pathways include UPR, mitochondrial dysfunction, lipid metabolism, and oxidative stress, many of these changes were predominantly pro-survival and adaptive mechanisms. The majority of the DRPs belong to ER and mitochondria demonstrating that these organelles play a key regulatory role in stress responses in oocytes.

In contrast to the changes in metabolic proteins in CCs, the relative abundance of the mitochondrial HADHB (complex I^32^), UQCRB (complex III) and CYCS were increased in oocytes suggesting an increased electron transport activity. This is matching with the increased MMP (JC1-aggregates). HADHB catalyzes three out of the four steps in FAO^32^. This increased bioenergetic activity may be a compensation for the reduced energy production and altered metabolism in CCs, as suggested by the proteomic changes and the reduced viability. However, ROS production was also increased in the PA-oocytes as shown by the increased CellRox staining. In addition, PA increased the relative abundance of few mitochondrial proteins with antioxidative functions, particularly, Peroxiredoxin 3 (PRDX3) which is a mitochondrion-specific H_2_O_2_-scavenging enzyme^42^. We noticed obvious co-localization of CellRox deep red staining in oocytes with mitochondria (JC1) suggesting that mitochondria are particularly the source of- and most affected by- the oxidative stress. This may also explain why the mitochondrial specific PRDX3 was specifically upregulated. PRDX3 is also required for mitochondrion organization and regulation of MMP^42^. Interestingly, PRDX3 is not only regulated by cellular redox status but also by acetylation. This can be linked with the reduced abundance of HINT2 in PA-treated oocytes, since HINT2 deletion provoked mitochondrial deformities and changed the pattern of acetylation in Hela cells^43^. PA-exposure during IVM has been recently reported to increase mitochondrial protein hyperacetylation patterns in porcine oocytes^44^.

In addition to the evident increase in metabolic activity in the oocyte, pathways related to amino acid metabolism were also significantly affected; ↑QDPR, a cofactor for phenylalanine, tyrosine, and tryptophan hydroxylases, suggests high rates of amino acid degradation. Amino acid degradation is an immediate cellular response to oxidative stress^45^. Inheritance of sensitized mitochondria with increased metabolic rate and high amino acid turnover from a metabolically-compromised oocyte to the embryo may contribute to the pathogenesis of reduced subsequent embryo development, since the most viable preimplantation embryos were shown to have the lowest metabolic rates, lowest glycolytic rates and/or lowest amino acid turnover (which is known as the “quiet embryo metabolism hypothesis”^46^).

Importantly, MAPK2K2 appears to play a key role in mediating PA-induced cellular stress in oocytes. Several altered canonical pathways involve MAPK2K2, namely, p70S6K Signaling, EIF2 Signaling, PI3K/AKT Signaling and NRF2-mediated Oxidative Stress Response. Both p70S6K and EIF2 signaling (regulated by mTOR) regulate cell survival, protein synthesis, autophagy, and transcription^47^. Therefore, these cellular modifications appear to be employed in oocytes to support oocyte viability and increase resistance to lipotoxicity. p70S6K signaling is the top canonical pathway affected in oocytes, which beside interacting with eIF2 pathways for regulation of translation and RNA processing, promotes cell survival through 14-3-3-mediated signaling^48^. This can be facilitated by the upregulated 14-3-3 protein gamma (YWHAG) noted here. NRF2-mediated Oxidative Stress Response has been recently shown to mediate bovine embryo survival under oxidative-stress conditions^49^. In addition to MAPK2K2, GSR and STIP1, members of NRF2-mediated response, were upregulated in the PA-treated oocytes. Mouse models with enhanced Nrf2 activation were shown to have increased transcription of *Gsr* that is involved in GSH biosynthesis and regeneration^50^. The increase in QDPR may also contribute to the oocyte’s defense mechanism against oxidative stress since it was shown to induce *SOD1* and *GPX3* expression kidney cells^51^.

This network of regulatory molecules is further enriched by the fact that both HSPA8 and its co-chaperon, STIP1^52^ are upregulated in PA-oocytes. Biological functions of HSPA8 (see Supplementary Table 1c) involve chaperone cofactor-dependent protein refolding, autophagy, regulation of mRNA stability, which may support oocyte survival. Other UPR proteins also increased in the PA-oocytes, such as those involved in ER stress pathway (↑P4HB, but ↓HSPA5), chaperon mediated protein folding (↑CCT8, ↑FKBP4), protein refolding and regulation of protein stability (↑HSPA8), ubiquitin dependent protein catabolic process (↑COPS3, ↑BCAP31, ↑ELOB), as well as lysosomal chaperon-mediated autophagy (↑HSPA8, ↑CTTN). Negative regulators of apoptosis were also upregulated in the affected oocytes, namely AKR1B1, MYDGF, P4HB, CTTN. Looking further into the data, we found that mitochondrial UPR markers HSPD1 (FC = 1.22, *P* = 0.065) and HSPE1 (FC = 1.62, *P* = 0.09) both tended to be upregulated which may suggest that mitochondrial UPR is also involved^18^.

Finally, the increased abundance of Cytochrome C (CYCS) in the PA-treated oocytes is alarming. Besides its role in energy production, CYCS regulates induction of apoptosis. However, here this should not be necessarily interpreted as an evidence of apoptosis in PA-treated oocytes, especially considering that the majority of these oocytes were developmentally competent. CYCS induce apoptosis when released into the cytosol due to outer mitochondrial membrane (OMM) permeabilization caused by high levels of oxidative and mitochondrial stress^53^. PA-treated oocytes exhibit ↑SLC24A5, a negative regulator of OMM permeabilization which may prevent apoptosis and support other pro-survival mechanisms in the oocyte. Nevertheless, it is important to consider that the ability of an oocyte to maintain survival under metabolic stress conditions may be reduced later during early embryo development since fertilization may exaggerate intracellular ROS levels^54^. High oxidative stress during early cleavage stage have been shown to increase embryo fragmentation, which is also noticed here in embryos derived from PA-treated oocytes. Treatments aiming to reduce cellular stress levels at this stage can be thus useful to increase survival during early embryo development.

Collectively, these data show evidence that cumulus-enclosed oocytes are sensitive to lipotoxic changes in their microenvironment. These changes were detected by shotgun proteomics and provide a unique, very informative insight into the oocyte’s response mechanisms to metabolic stress compared with the surrounding CCs. These changes are detectable following a short-term exposure during IVM, which mimics a post-LH phase. The magnitude of these adaptive mechanisms may differ under *in vivo* conditions where oocytes undergo longer exposure to metabolic stress during follicular growth. Extrapolation of these data to the *in vivo* settings should therefore be done with caution. We have previously described longer-term exposure effects of FFAs during *in vitro* murine follicular growth and maturation for 13 days which had similar detrimental impact on oocyte developmental competence compared to short term exposure^10^.

In conclusion, while DRPs in CCs suggest metabolic failure and pro-apoptotic signaling, proved by mitochondrial dysfunction and increased apoptosis, the enclosed oocytes undergo detectable proteomic compensatory metabolic adaptations and responses to oxidative stress that were predominantly pro-survival. Clearly, UPRer and mitochondrial proteins play an important regulatory role in these adaptive responses in oocytes. In addition, several other prominent proteins appear to contribute, such as PRDX3, HADHB, MAPK2K2, STIP1-HSPA8, as well as the NRF2-mediated oxidative stress response, p70S6K and 14-3-3 mediated signaling. The majority of these responses have not been previously studied in oocytes. Nevertheless, high levels of CYCS and oxidative stress makes these oocytes vulnerable during subsequent development if not handled under supportive conditions. These data enriches the current understanding of the mechanisms by which the oocyte can respond to metabolic stress and forms a fundamental base for subsequent studies to focus on the specific role of each of these pathways in metabolically-compromised oocytes. The study also highlights avenues through which the developmental capacity of metabolically-compromised oocytes may be improved, protected or rescued.

## Materials and Methods

### Collection of cumulus oocyte complexes

Bovine ovaries from a local abattoir were transferred to our laboratory within 2 h of slaughter in warm saline. Ovaries were washed and visible antral follicles (2–6 mm in diameter) were aspirated. Follicular fluid was allowed to set for 5 min and the cellular precipitate was transferred to a searching dish containing Hepes-buffered Wash-TALP media as described by Van Hoeck, *et al*.^11^. Good quality COCs (with dark, homogenous ooplasm and more than 5 layers of compact cumulus cells) were selected and used in the experiments.

### *In vitro* maturation and treatments

Stock solutions of PA (150 mM) were prepared in ethanol. Maturation media was composed as described earlier^13^ based on TCM-199 media supplemented with 0.75%w/v FA-free BSA as a carrier for PA, and murine epidermal growth factor (mEGF, 20 ng/ml) to stimulate oocyte maturation. Selected COCs were washed and cultured in groups of 50 ± 5 COCs in 4-well plates containing maturation media (500 µL/well) supplemented with PA (150 µM) or solvent (ethanol, 0.01%) for 24 h in 5% CO_2_ in humidified air at 38.5 °C. This PA concentration is pathophysiologically relevant considering the elevated PA concentration in FF in obese women^55^ and in cows during negative energy balance^56^. COCs were then either fertilized and further cultured to examine subsequent developmental competence, or separated into CCs and oocytes and snap-frozen for proteomic analysis, or used for other functional parameters to assess oocyte quality and cumulus cell viability.

### Immunohistochemical determination of apoptosis in COCs

At 22 h of IVM, PA and solvent COCs (n = 69 from 3 replicates) were fixed in paraformaldehyde 4% for 1 h then stored in PBS containing PVP (1 mg/mL) at 4 °C. Cumulus cell apoptosis was determined by immunostaining with rabbit-anti-cleaved Caspase-3 (CASP3, Asp 175, Cell Signaling Technology, 1:250) as previously described^12^. Images were obtained using a fluorescence Olympus microscope IX71. The percentage of positively stained cells were determined using ImageJ as described by Jensen^57^ within each COC. COCs were categorized according to the percentage of apoptotic cells (as 0–5%, 5–10%, 10–25%, 25–50%, >50%).

### Assessment of mitochondrial activity and intracellular reactive oxygen species in COCs

A combined fluorescence staining technique using 5,5′,6,6′-tetrachloro-1,1′,3,3′-tetraethyl-benzimidazolyl-carbocyanine iodide (JC-1, Invitrogen) and CellROX™ Deep Red Reagent (ThermoFisher) were used (as validated and described earlier by De Biasi, *et al*.^58^ and Komatsu, *et al*.^59^) to estimate mitochondrial inner membrane potential (MMP) and oxidative stress, respectively, in the oocytes and surrounding CCs. In 3 replicates, Control and PA COCs were transferred at 22 h of IVM to a Petri dish in a droplet of Wash-TALP media at 38 °C on a warm-stage. COCs (n = 42) were denuded using EZ-tip (125 µm) attached to an EZ-Grip micropipettor (Origio). Other COCs (n = 46) were used for imaging of CCs. Oocytes and COCs were incubated in maturation media containing JC1 (5 µg/mL) and CellRox deep red (2.5 mM) (from 1000X stock solutions in DMSO) for 30 min at 5% CO_2_ and 38.5 °C. They were then washed and kept in wash-TALP droplets under oil in a 35 mm dish with glass bottom and immediately examined under a Leica SP8 confocal microscope equipped with white laser source (Leica WLL) lasers at excitation/emission 488/525 nm (to detect JC-1 monomers; green), 561/590 nm (JC1-aggregates; yellow) and 644/665 nm (to visualize CellRox staining). The microscope was enclosed in a humid chamber adjusted at 37 °C. One optical section was examined for each oocyte at the pericortical area of the oocyte or at the level of CCs. The grey scale intensity in each channel was measured using Leica Application Suite X (LAS X) software. Florescence intensities in oocytes were determined only in the area of the ooplasm, i.e. excluding the zona pellucida which may contain remnants of mitochondria from the CC extrusions. MMP was calculated as the ratio of the grey scale intensity at 590 nm:525 nm. Oxidative stress was estimated based on the grey scale intensity at 665 nm.

### *In vitro* fertilization and embryo culture

A total of 603 COCs in four replicates were used to study developmental competence. For that, at 24 h of IVM, COCs were fertilized (in Fert-TALP medium, groups of 100 ± 10 COCs, 22 h) and cultured (in mSOF, groups of 25 ± 5 embryos, 7 days) as previously described^12^. Sample size calculation for the number of COCs used to determine developmental competence was calculated based on a power of 80% to detect a 10% difference in blastocyst rates between the PA and solvent groups at *P* = 0.05 (PS: Power and Sample Size Calculation version 3.1.6, Vanderbilt Biostatistics). At 48 h pi, proportions of cleaved embryos, good quality embryos (≥4-cells) and those with >15% fragmentation of cellular mass were recorded. Blastocyst rates (8 days p.i.) were recorded and expressed as a proportion of total number of oocytes. Morphological classification was done under an inverted Olympus CKX41 microscope (Olympus, Aartselaar, Belgium). Blastocysts were fixed on Day 8 in 4% paraformaldehyde (30 min) and stored in PBS containing PVP (1 mg/ml) for quality assessment using differential immunostaining staining with CASP3 antibody (apoptosis), anti-CDX2 antibody (for trophectoderm cell count), and Hoechst (for total cell count) as previously described^12^.

### Proteomic analysis of CCs and oocytes

A TMT shotgun-MS proteomic analyses was performed on protein extracts of pools of 120–200 oocytes or their corresponding CCs (3 replicates). Sample preparation, protein extraction, digestion and TMT labelling, and Nano reversed phase liquid chromatography and mass spectrometry were done as described in supplementary file (Appendix 1). Spectra were analyzed in Proteome discoverer (2.1) software against Bos Taurus database using only medium and high confident peptides with a global FDR <5% based on a target-decoy approach^60^ and normalization was done by the CONSTANd algorithm^61^ (see Appendix 1 for more details). Differentially regulated proteins (DRPs) were determined based on a 5% FDR, adjusted *P* value <0.05, and more than 10% fold change in PA compared to control groups. Heat maps and hierarchical clustering of the proteomics data were performed using MultiExperimentViewer (MeV) version 4.9.0 (Bio-Soft Net). GO-Cellular component analysis was performed in Panther classification system (Panther13.1) and by manual extraction of subcellular location data from the UniProt database if available (uniprot.org). Canonical pathways of the DRPs and comparison analysis between PA effects in CCs and oocytes were performed using Core Analyses in Ingenuity® Pathway Analysis (IPA, QIAGEN bioinformatics).

### Statistical analysis

Cleavage and blastocyst rates were analysed using a binary logistic regression models in IBM SPSS Statistics 23 (for Windows, Chicago, IL, USA). Numerical data e.g. from JC1 staining and CellRox quantification were analysed using linear mixed models. In all analyses, replicates, treatments and their interactions were taken into account. The interactions had no significant effects and were omitted from the models. A Bonferroni post-hoc test was performed to correct for multiple comparisons. All data were from at least three independent replicates. Differences of *P* values ≤ 0.05 were considered significant and those >0.05 and ≤0.1 were reported as tendencies.

## Supplementary information

Supplementary information

## Data Availability

The datasets generated and analyzed during the current study are available from the corresponding author on reasonable request. Proteomics data are available via ProteomeXchange with identifier PXD012141.

## References

[1] Jungheim ES, Travieso JL, Carson KR, Moley KH (2012). Obesity and reproductive function. Obstet Gynecol Clin.

[2] Jungheim ES (2011). Associations between free fatty acids, cumulus oocyte complex morphology and ovarian function during *in vitro* fertilization. Fertil Steril.

[3] Pandey S, Pandey S, Maheshwari A, Bhattacharya S (2010). The impact of female obesity on the outcome of fertility treatment. J Hum Reprod Sci.

[4] Luke B (2011). Female obesity adversely affects assisted reproductive technology (ART) pregnancy and live birth rates. Hum Reprod.

[5] Cardozo ER, Karmon AE, Gold J, Petrozza JC, Styer AK (2016). Reproductive outcomes in oocyte donation cycles are associated with donor BMI. Hum Reprod.

[6] Leroy JL (2011). Intrafollicular conditions as a major link between maternal metabolism and oocyte quality: a focus on dairy cow fertility. Reprod Fertil Dev.

[7] Valckx SD (2012). BMI-related metabolic composition of the follicular fluid of women undergoing assisted reproductive treatment and the consequences for oocyte and embryo quality. Hum Reprod.

[8] Mirabi P (2017). The role of fatty acids on ICSI outcomes: a prospective cohort study. Lipids Health Dis.

[9] Shimabukuro M (2006). Metabolic syndrome and lipotoxicity. *Nihon Naika Gakkai zasshi*. The Journal of the Japanese Society of Internal Medicine.

[10] Valckx SD (2014). Elevated non-esterified fatty acid concentrations during *in vitro* murine follicle growth alter follicular physiology and reduce oocyte developmental competence. Fertil Steril.

[11] Van Hoeck V (2011). Elevated non-esterified fatty acid concentrations during bovine oocyte maturation compromise early embryo physiology. PLoS One.

[12] Marei WFA (2017). Alpha-linolenic acid protects the developmental capacity of bovine cumulus-oocyte complexes matured under lipotoxic conditions *in vitro*. Biol Reprod.

[13] Desmet KL (2016). Exposure of bovine oocytes and embryos to elevated non-esterified fatty acid concentrations: integration of epigenetic and transcriptomic signatures in resultant blastocysts. BMC Genomics.

[14] Lolicato F (2015). The cumulus cell layer protects the bovine maturing oocyte against fatty acid-induced lipotoxicity. Biol Reprod.

[15] Weinberg JM (2006). Lipotoxicity. Kidney Int.

[16] Zhang K, Kaufman RJ (2008). From endoplasmic-reticulum stress to the inflammatory response. Nature.

[17] Sutton-McDowall ML (2016). Nonesterified Fatty Acid-Induced Endoplasmic Reticulum Stress in Cattle Cumulus Oocyte Complexes Alters Cell Metabolism and Developmental Competence. Biol Reprod.

[18] Munch C, Harper JW (2016). Mitochondrial unfolded protein response controls matrix pre-RNA processing and translation. Nature.

[19] Runkel ED, Baumeister R, Schulze E (2014). Mitochondrial stress: balancing friend and foe. Exp Gerontol.

[20] Schatten H, Sun QY, Prather R (2014). The impact of mitochondrial function/dysfunction on IVF and new treatment possibilities for infertility. Reprod Biol Endocrinol.

[21] Campbell BK (2003). Domestic ruminants as models for the elucidation of the mechanisms controlling ovarian follicle development in humans. Reprod Suppl.

[22] Van Hoeck V (2013). Oocyte developmental failure in response to elevated nonesterified fatty acid concentrations: mechanistic insights. Reproduction.

[23] Miura Y, Endo T (2010). Survival responses to oxidative stress and aging. Geriatr Gerontol Int.

[24] Aardema H (2011). Oleic acid prevents detrimental effects of saturated fatty acids on bovine oocyte developmental competence. Biol Reprod.

[25] Marei WF, Wathes DC, Fouladi-Nashta AA (2012). Differential effects of linoleic and alpha-linolenic fatty acids on spatial and temporal mitochondrial distribution and activity in bovine oocytes. Reprod Fertil Dev.

[26] Marei WF, Abayasekara DR, Wathes DC, Fouladi-Nashta AA (2014). Role of PTGS2-generated PGE2 during gonadotrophin-induced bovine oocyte maturation and cumulus cell expansion. Reproductive biomedicine online.

[27] Marei WFA (2017). Effect of nutritionally induced hyperlipidaemia on *in vitro* bovine embryo quality depends on the type of major fatty acid in the diet. Reprod Fertil Dev.

[28] De Bie, J. *et al*. Differential effects of high and low glucose concentrations during lipolysis-like conditions on bovine *in vitro* oocyte quality, metabolism and subsequent embryo development. *Reprod Fertil Dev*, 10.1071/RD16474 (2017). 10.1071/RD16474 28390473

[29] De Bie J (2017). Differential effects of high and low glucose concentrations during lipolysis-like conditions on bovine *in vitro* oocyte quality, metabolism and subsequent embryo development. Reprod Fertil Dev.

[30] Abdel-Haleem AM (2017). The Emerging Facets of Non-Cancerous Warburg Effect. Frontiers in endocrinology.

[31] Ramana KV (2011). ALDOSE REDUCTASE: New Insights for an Old Enzyme. Biomol Concepts.

[32] Wang Y, Mohsen A-W, Mihalik SJ, Goetzman ES, Vockley J (2010). Evidence for Physical Association of Mitochondrial Fatty Acid Oxidation and Oxidative Phosphorylation Complexes. The Journal of biological chemistry.

[33] Lefort N (2010). Increased Reactive Oxygen Species Production and Lower Abundance of Complex I Subunits and Carnitine Palmitoyltransferase 1B Protein Despite Normal Mitochondrial Respiration in Insulin-Resistant Human Skeletal Muscle. Diabetes.

[34] Kozjak-Pavlovic V (2017). The MICOS complex of human mitochondria. Cell and tissue research.

[35] Luzzo KM (2012). High fat diet induced developmental defects in the mouse: oocyte meiotic aneuploidy and fetal growth retardation/brain defects. PLoS One.

[36] Marciniak SJ (2004). CHOP induces death by promoting protein synthesis and oxidation in the stressed endoplasmic reticulum. Genes Dev.

[37] Harding HP (2000). Regulated translation initiation controls stress-induced gene expression in mammalian cells. Mol Cell.

[38] Moore K, Hollien J (2015). Ire1-mediated decay in mammalian cells relies on mRNA sequence, structure, and translational status. Mol Biol Cell.

[39] Hollien J, Weissman JS (2006). Decay of endoplasmic reticulum-localized mRNAs during the unfolded protein response. Science.

[40] Mu YM (2001). Saturated FFAs, palmitic acid and stearic acid, induce apoptosis in human granulosa cells. Endocrinology.

[41] Aardema H (2013). Bovine cumulus cells protect maturing oocytes from increased fatty acid levels by massive intracellular lipid storage. Biol Reprod.

[42] Chang T-S (2004). Peroxiredoxin III, a Mitochondrion-specific Peroxidase, Regulates Apoptotic Signaling by Mitochondria. Journal of Biological Chemistry.

[43] Martin J (2013). Disruption of the histidine triad nucleotide-binding hint2 gene in mice affects glycemic control and mitochondrial function. Hepatology.

[44] Itami, N., Shirasuna, K., Kuwayama, T. & Iwata, H. Palmitic acid induces ceramide accumulation, mitochondrial protein hyper-acetylation and mitochondrial dysfunction in porcine oocytes. *Biol Reprod*, 10.1093/biolre/ioy023 (2018). 10.1093/biolre/ioy023 29385411

[45] Pacifici, R. E. & Davies, K. J. A. In *Methods in Enzymology* Vol. 186, 485–502 (Academic Press, 1990). 10.1016/0076-6879(90)86143-j 2233315

[46] Leese HJ (2002). Quiet please, do not disturb: a hypothesis of embryo metabolism and viability. Bioessays.

[47] Gutierrez-Uzquiza A, Arechederra M, Bragado P, Aguirre-Ghiso JA, Porras A (2012). p38alpha mediates cell survival in response to oxidative stress via induction of antioxidant genes: effect on the p70S6K pathway. The Journal of biological chemistry.

[48] Lim GE, Piske M, Johnson JD (2013). 14-3-3 proteins are essential signalling hubs for beta cell survival. Diabetologia.

[49] Amin A (2014). Bovine embryo survival under oxidative‐stress conditions is associated with activity of the NRF2‐mediated oxidative‐stress‐response pathway. Molecular reproduction and development.

[50] Wu KC, Cui JY, Klaassen CD (2011). Beneficial Role of Nrf2 in Regulating NADPH Generation and Consumption. Toxicological Sciences.

[51] Gu Y-t (2017). Protective effect of dihydropteridine reductase against oxidative stress is abolished with A278C mutation. *Journal of Zhejiang University*. Science. B.

[52] Woodford MR (2016). The FNIP co-chaperones decelerate the Hsp90 chaperone cycle and enhance drug binding. Nature communications.

[53] Liu X, Kim CN, Yang J, Jemmerson R, Wang X (1996). Induction of apoptotic program in cell-free extracts: requirement for dATP and cytochrome c. Cell.

[54] Lopes AS, Lane M, Thompson JG (2010). Oxygen consumption and ROS production are increased at the time of fertilization and cell cleavage in bovine zygotes. Hum Reprod.

[55] Valckx SD (2014). Fatty acid composition of the follicular fluid of normal weight, overweight and obese women undergoing assisted reproductive treatment: a descriptive cross-sectional study. Reprod Biol Endocrinol.

[56] Leroy JL (2005). Non-esterified fatty acids in follicular fluid of dairy cows and their effect on developmental capacity of bovine oocytes *in vitro*. Reproduction.

[57] Jensen EC (2013). Quantitative Analysis of Histological Staining and Fluorescence Using Image J. The Anatomical Record.

[58] De Biasi, S., Gibellini, L. & Cossarizza, A. Uncompensated Polychromatic Analysis of Mitochondrial Membrane Potential Using JC-1 and Multilaser Excitation. *Current protocols in cytometry* **72**, 7.32.31-11, 10.1002/0471142956.cy0732s72 (2015). 10.1002/0471142956.cy0732s72 25827483

[59] Komatsu K (2014). Mitochondrial membrane potential in 2-cell stage embryos correlates with the success of preimplantation development. Reproduction.

[60] Nesvizhskii AI, Vitek O, Aebersold R (2007). Analysis and validation of proteomic data generated by tandem mass spectrometry. Nature methods.

[61] Maes E (2016). CONSTANd: A Normalization Method for Isobaric Labeled Spectra by Constrained Optimization. Mol Cell Proteomics.

