# Supplementary material for: Proteomic changes in oocytes after in vitro maturation in lipotoxic conditions are different from those in cumulus cells
Source: Sci Rep. 2019 Mar 6;9:3673. doi: 10.1038/s41598-019-40122-7 (PMC6403224; doi:10.1038/s41598-019-40122-7)
Supplement: Supplementary file 1 — Supplementary information [file 41598_2019_40122_MOESM1_ESM.pdf]

Appendix I: Proteomic analysis of CCs and oocytes

Sample preparation

Pools of denuded oocytes and their corresponding CCs (collected from 120-200 COCs per replicate, 3 replicates) were used for the analysis. Samples were lysed using 25 µL RIPA buffer (1x) (Thermo Scientific, Rockford, IL) containing 1x HALT phosphatase inhibitor (Thermo Scientific) and 1x HALT protease inhibitor (Thermo Scientific), combined with a 30 s sonication (Branson Sonifier SLPe ultrasonic homogenizer, Labequip, Ontario, Canada) on ice. After centrifugation of the samples for 15 min at 14,000 *g* on 4°C, the pellet was discarded. To improve further solubilisation of the proteins and to provide an efficient digestion, 0.1% Rapigest SF surfactant (Waters, Milford, MA) was added to the supernatant and the sample was incubated for 5 min at 100 °C. Next, the protein concentration was determined using the Pierce BCA protein Assay kit (Thermo Scientific).

Digestion and TMT labelling

Before labelling the samples, 9.5 µg protein of each sample was reduced using 1.25 µl of 500 mM tris(2-carboxyethyl) phosphine (TCEP), supplied with the TMT labeling kit (Thermo Scientific), in a volume of 100 µl of 100 mM TEAB, and incubated for 1 h at 55°C. Next, the samples were alkylated with iodoacetamide in dark at room temperature and digested overnight at 37°C with trypsin (enzyme:protein ratio = 1:20). Afterwards, the tryptic digests were desalted using Pierce C18 spin columns (Thermo Scientific) according to manufacturer's instructions. Next, the eluted peptides were vacuum dried and reconstituted in 100mM TEAB to a final concentration of 1µg/µl before labelling was performed.

For the reconstitution of the tags, the TMT labels were dissolved in 41 µl acetonitrile according to the manufacturer's protocol. From every sample, 5 µg was labeled with 4 µl of a TMT tag dissolved in acetonitrile and every sample was incubated for 1 hour at ambient temperature.

Labels 126-128 were used to label the 3 replicates of control oocyte samples, while labels 129-131 were used for the 3 replicates of the PA-treated oocyte samples. The same was done for the cumulus cell samples. The labelling reaction was stopped by adding 2µl 5% hydroxylamine. After 15 minutes, a pooled sample was prepared based on the six labelled samples with a protein concentration ratio of 1:1:1:1:1:1. To remove the excess of labels, the pooled sample was cleaned again using C18 spin columns and vacuum dried.

Nano reversed phase liquid chromatography and mass spectrometry

Peptide mixtures were separated by reversed phase C18 chromatography on an Easy nanoLC 1000 system using an Acclaim C18 PepMap®100 column (75 µm x 2 cm, 3 µm particle size) connected to an Acclaim PepMap™ RSLC C18 analytical column (50 µm x 15 cm, 2 µm particle size) (Thermo Scientific, San Jose, CA). Before loading, pellets were dissolved in mobile phase A, containing 2% acetonitrile and 0.1% formic acid, and spiked with 20 fmol Glu-1-fibrinopeptide B (Glu-fib, Protea biosciences, Morgantown, WV). For each TMT experiment 1µg of labelled peptides (in a volume of 10µL) were loaded on the column. A linear gradient of mobile phase B (0.1% formic acid in 95% acetonitrile) from 2 to 45% in 230 min. followed by a steep increase to 100% mobile phase B in 10 min was used at a flow rate of 300 nL/min.
Liquid Chromatography was followed by MS (LC-MS/MS) and was performed on a Q-Exactive Plus mass spectrometer equipped with a Nanospray Flex ion source (Thermo Fisher, Waltham, MA, USA). The high resolution orbitrap mass spectrometer was set up in a MS/MS mode where a full scan spectrum (350 – 1850 m/z, resolution 70,000) was followed by a maximum of five high energy collision activated dissociation (HCD) tandem mass spectra (100 to 2000 m/z). Peptide ions were selected for further interrogation by tandem MS as the five most intense peaks of a full scan mass spectrum. The normalized collision energy used was set at 35%. A dynamic exclusion list of 15 sec for data dependent acquisition was applied.

Data analysis

Proteome discoverer (2.1) software (Thermo Scientific, San Jose, CA) was used to perform database searching against the bos taurus reference database, generated from Uniprot (UP000009136, 23971 entries) using both the SEQUEST and Mascot algorithms. The following settings were applied: precursor mass tolerance of 10 ppm, fragment mass tolerance of 0.02 Da. Trypsin was specified as digesting enzyme and 2 missed cleavages are allowed. Cysteine carbamidomethylation and TMT modifications (N-terminus and lysine residues) were defined as fixed modifications and methionine oxidation and phosphorylation (STY) were variable modifications. The results were filtered using following settings: only medium and high confident peptides with a global FDR < 5% based on a target-decoy approach (28) and first ranked peptides were included in the results. In the TMT quantitation workflow the most confident centroid method was used with an integration window of 20 ppm. The extracted reporter ion intensities of all the quantification channels in all the runs were transformed by the logarithmic function with base 2. The log_2-transformed intensity distributions were normalized by the CONSTANd algorithm as described by Maes*, et al.* (29). Differentially regulated proteins (DRPs) were determined based on a

5% FDR, adjusted *P* value < 0.05, and more than 10% fold change in PA compared to control groups. Heat maps and hierarchical clustering of the proteomics data were performed using MultiExperimentViewer (MeV) version 4.9.0 (Bio-Soft Net). GO-Cellular component analysis was performed in Panther classification system (Panther13.1) and by manual extraction of subcellular location data from the UniProt database if available (uniprot.org). Canonical pathways of the DRPs and comparison analysis between PA effects in CCs and oocytes were performed using Core Analyses in Ingenuity® Pathway Analysis (IPA, QIAGEN bioinformatics).

Appendix II

Supplementary table 1a. Proteins with increased abundance due to PA exposure in CCs and their GO biological processes.

**Symbol; Entrez Gene Name (UniProt Accession)**	**FC**	**Expr p-value**	**Subcellular location**	**GO Biological Process**	
**SCO1;** cytochrome c oxidase assembly protein, mitochondrial (A1A4J8)	6.56	3,89E-02	Mit.	• copper ion transport<br>• Mitochondrial respiratory chain complex IV assembly	• negative regulation of proteasomal protein catabolic process
**ATAD1**; ATPase family, AAA domain containing 1 (Z4YH78)	3.51	4,66E-02	PM and Mit.	• positive regulation of receptor internalization	
**FAF2;** Fas associated factor family member 2 (Q2HJD0)	1.73	4,99E-02	ER and lipid droplets	• lipase inhibitor activity<br>• lipid particle organization<br>• retrograde protein transport, ER to cytosol	• response to unfolded protein<br>• ubiquitin-dependent ERAD pathway
**EIF4A2;** eukaryotic translation initiation factor 4A2 (Q3SZ65)	1.67	9,02E-03	Cytoplasm, Nucleus	• cellular response to leukemia inhibitory factor<br>• negative regulation of RNA-directed 5'-3' RNA polymerase activity	• regulation of gene expression<br>• RNA secondary structure unwinding<br>• translational initiation
**MYO1B;** myosin IB (A6QLD6)	1.49	4,29E-02	early endosome, golgi	• actin filament-based movement	• post-Golgi vesicle-mediated transport
**TUBA1A;** tubulin alpha 1a (P81947)	1.48	4,88E-02	Cytoskeleton	• microtubule-based process	
**TKFC;** triokinase and FMN cyclase (A0JN77)	1.47	4,03E-02	Cytoplasm	• carbohydrate phosphorylation<br>• glycerol metabolic process	• negative regulation of MDA-5 signaling pathway
**EEF1G;** eukaryotic translation elongation factor 1 gamma (F1MG05)	1.45	3,07E-07	ER	• translation elongation factor activity<br>• cadherin binding	•
**RPL6;** ribosomal protein L6 (Q58DQ3)	1.43	7,61E-03	Rough ER	• RNA binding<br>• cytoplasmic translation	• ribosomal large subunit assembly
**PRMT1;** protein arginine methyltransferase 1 (Q5E949)	1.40	9,36E-04	Nucleoplasm	• histone-arginine N-methyltransferase activity<br>• histone H4-R3 methylation	• protein methylation<br>• regulation of transcription, DNA-templated

**RPS7;** ribosomal protein S7 (A6H769)	1.33	3,28E-02	centrosome ribosome	• cytoplasmic translation	• ribosomal small subunit biogenesis<br>• rRNA processing
**RPS24;** ribosomal protein S24 (Q56JU9)	1.32	4,19E-02	small ribosomal subunit	• maturation of SSU-rRNA from tricistronic rRNA transcript (SSU-rRNA, 5.8S rRNA, LSU-rRNA) and translation	
**GSTM3;** glutathione S-transferase mu 3 (Q2KIV8)	1.32	4,03E-02	Cytoplasm	• glutathione transferase activity	• metabolic process
**RPS3;** ribosomal protein S3 (Q3T169)	1.31	4,03E-02	Cytoskeleton, Membrane, Mit., MIM, Nucleus	• DNA damage response, detection of DNA damage<br>• damaged DNA binding and negative regulation of DNA repair<br>• positive regulation of intrinsic apoptotic signaling pathway in response to DNA damage<br>• cellular response to hydrogen peroxide	• Hsp70 and hsp90 protein binding<br>• negative regulation of protein ubiquitination positive regulation of gene expression<br>• negative regulation of translation cellular response to tumor necrosis factor<br>• positive regulation of NF-kappaB transcription factor activity
**PABPC4;** poly(A) binding protein cytoplasmic 4 (A4IFC3)	1.31	1,27E-02	Cytoplasm	• mRNA binding	• poly(A) binding and regulation of mRNA stability
**FUS;** RNA-binding protein FUS (Q28009)	1.31	4,69E-02	Nucleus	• DNA binding	• regulation of transcription, DNA-templated
**LDHB;** lactate dehydrogenase B (B0JYN3)	1.31	1,63E-02	Cytoplasm	• carbohydrate metabolic process	• carboxylic acid metabolic process
**PGM2;** phosphoglucomutase 2 (A6QQ11)	1.31	7,04E-03	Cytoplasm	• glucose metabolic process	
**DNPEP;** aspartyl aminopeptidase (Q2HJH1)	1.30	7,61E-03	Cytoplasm	• peptide metabolic process	
**CSDE1**; cold shock domain containing E1 (A5D7D3)	1.30	3,81E-02	Cytoplasm	• regulation of transcription, DNA-templated	
**TALDO1;** transaldolase 1 (G5E5C8)	1.29	9,23E-03	Cytoplasm Nucleoplasm	• carbohydrate metabolic process<br>• pentose-phosphate shunt	
**MX1;** MX dynamin like GTPase 1 (I3QQD7)	1.29	4,99E-02	Cytoplasm	• GTPase activity<br>• GTP binding	
**AKR1B1**; aldo-keto reductase family 1 member B (Q5E962)	1.27	1,63E-02	Cytoplasm Nucleoplasm	• oxidoreductase activity	• negative regulation of apoptotic process
**PGK1**; phosphoglycerate kinase 1 (Q3T0P6)	1.27	6,01E-03	Cytoplasm	• cellular response to hypoxia	• glycolytic process

**CAP1;** cyclase associated actin cytoskeleton regulatory protein 1 (A6QLB7)	1.24	9,23E-03	PM, cytoskeleton	• receptor-mediated endocytosis<br>• actin cytoskeleton organization	
**COPA;** coatomer protein complex subunit alpha (Q27954)	1.23	4,39E-03	Cytoplasmic vesicle, Golgi .	• ER to Golgi vesicle-mediated transport<br>• intracellular protein transport	• intra-Golgi vesicle-mediated transport<br>• retrograde vesicle-mediated transport, Golgi to ER
**LDHA;** lactate dehydrogenase A (P19858)	1.23	2,83E-02		• carbohydrate metabolic process<br>• carboxylic acid metabolic process	
**SPTAN1;** spectrin alpha, non-erythrocytic 1 (E1BFB0)	1.23	4,03E-02	PM, , cytoskeleton	• calcium ion binding<br>• cadherin binding	
**UAP1;** UDP-N-acetylglucosamine pyrophosphorylase 1 (F1MJP7)	1.22	3,58E-02	PM, Cytoplasm,	• UDP-N-acetylglucosamine biosynthetic process	
**EZR;** ezrin (P31976)	1.21	1,77E-02	PM	• cortical microtubule organization<br>• negative regulation of ERK1 and ERK2 cascade<br>• negative regulation of p38MAPK cascade<br>• negative regulation of transcription from RNA polymerase II promoter<br>• positive regulation of gene expression	• phosphatidylinositol-mediated signaling<br>• positive regulation of cellular protein catabolic process<br>• positive regulation of early endosome to late endosome transport<br>• positive regulation of protein localization to early endosome
**PSMA6**; proteasome subunit alpha 6 (G5E5C3)	1.21	2,93E-02	Cytoplasm Nucleus	• positive regulation of NF-kappaB transcription factor activity	• ubiquitin-dependent protein catabolic process
**EEF2;** eukaryotic translation elongation factor 2 (Q3SYU2)	1.20	1,94E-03	Cytoplasm Nucleus	• translation elongation factor activity	• positive regulation of translation
**CLTC;** clathrin heavy chain (F1MPU0)	1.20	1,35E-04	PM, Cytoplasmic vesicles	• clathrin coat assembly<br>• intracellular protein transport	• vesicle-mediated transport
**PKM;** pyruvate kinase M1/2 (Q3ZC87)	1.19	2,97E-03	Cytoplasm	• pyruvate kinase activity<br>• Glycolysis	•
**PGD;** phosphogluconate dehydrogenase (Q3ZCI4)	1.17	3,63E-02	Cytoplasm	• D-gluconate metabolic process<br>• pentose biosynthetic process	• pentose-phosphate shunt, oxidative branch

Supplementary table 1b. Proteins with reduced abundance due to PA exposure in CCs and their GO biological processes.

Symbol; Entrez Gene Name (UniProt Accession)	Fold Change	Expr p-value	Subcellular location	GO Biological Process	
**HIST1H2BK;** histone cluster 1 H2B family member k (F1MUD2)	0.52	1,77E-02	Nucleus	• DNA binding<br>• protein heterodimerization activity	• nucleosome assembly
**CHCHD3;** coiled-coil-helix-coiled-coil-helix domain containing 3, MICOS complex subunit MIC19 (Q5E9D3)	0.57	4,03E-02	Cytoplasm, Membrane, Mit., MIM, Nucleus	• cristae formation<br>• mitochondrial fusion	• negative regulation of transcription from RNA polymerase II promoter transcription, DNA-templated
**B4GALT4;** beta-1,4-galactosyltransferase 4 (Q32LF7)	0.57	1,77E-02	Golgi apparatus	• carbohydrate metabolic process	•
**TIMM9;** translocase of inner mitochondrial membrane 9 (Q2KIV2)	0.63	2,18E-02	MIM	• chaperone-mediated protein transport	• protein import into mitochondrial inner membrane
**LMNB1;** lamin B1 (A7YY47)	0.64	1,63E-07	Cytoskeleton	• structural molecule activity	•
**CALU;** calumenin (F1N3H1)	0.65	2,90E-07	ER	• calcium ion binding	• Post-translational protein phosphorylation.
**SIL1;** nucleotide exchange factor (Q32KV6)	0.66	1,16E-02	ER lumen	• Protein transport, Translocation, Transport	•
**ATP5H;** ATP synthase, H+ transporting, mitochondrial Fo complex subunit D (P13620)	0.67	3,89E-05	Mit., MIM	• hydrogen ion transmembrane transporter activity<br>• ATP hydrolysis coupled cation transmembrane transport	• ATP synthesis coupled proton transport
**CALR;** calreticulin (A5D7J6)	0.68	2,54E-05	ER	• negative regulation of:<br>○ cell cycle arrest<br>○ intracellular steroid hormone receptor signaling pathway<br>○ retinoic acid receptor signaling pathway<br>○ transcription from RNA polymerase II promoter<br>○ translation<br>○ trophoblast cell migration	• positive regulation of<br>○ cell proliferation<br>○ DNA replication<br>○ gene expression<br>○ NIK/NF-kappaB signaling<br>• protein export from nucleus<br>• protein folding<br>• protein localization to nucleus<br>• protein stabilization<br>• cortical actin cytoskeleton organization

**P4HA1;** prolyl 4-hydroxylase subunit alpha 1 (A6QL77)	0.69	2,88E-06	ER and Mit.	• oxidoreductase activity	• peptidyl-proline hydroxylation to 4-hydroxy-L-proline
**CTSB;** cathepsin B (P07688)	0.70	4,99E-02	Lysosome	• proteolysis involved in cellular protein catabolic process	• regulation of catalytic activity
**MYEF2;** myelin expression factor 2 (A6QQP0)	0.70	4,10E-02	Nucleus	RNA polymerase II transcription factor activity, sequence-specific DNA binding and single-stranded DNA binding	
RCN1; reticulocalbin 1 (A4IF88)	0.72	3,17E-02	Cytoplasm	• calcium ion binding	•
**SCP2;** sterol carrier protein 2, Non-specific lipid-transfer protein (F1MV74)	0.72	4,19E-02	Mit.,Nucleus Peroxisome	• cholesterol binding<br>• oleic acid binding<br>• lipid hydroperoxide transport	• metabolic process<br>• peroxisome organization<br>• positive regulation of intracellular cholesterol transport
**PDIA3;** protein disulfide isomerase family A member 3 (A5D7E8)	0.72	1,20E-07	ER	• cell redox homeostasis	• positive regulation of extrinsic apoptotic signaling pathway
**PDIA6;** protein disulfide isomerase family A member 6 (A6QNL5)	0.72	1,49E-04	ER Cytoplasm	• apoptotic cell clearance<br>• cell redox homeostasis	• protein folding<br>• response to ER stress
**CISD1;** CDGSH iron sulfur domain 1 (Q3ZBU2)	0.73	3,27E-02	MOM,	• regulation of cellular respiration	•
**COX5A;** cytochrome c oxidase subunit 5A (P00426)	0.73	1,52E-02	MIM,	• cytochrome-c oxidase activity	• mitochondrial electron transport, cytochrome c to oxygen
**P4HB;** prolyl 4-hydroxylase subunit beta (A6H7J6)	0.73	4,26E-09	ER	• cell redox homeostasis<br>• cellular response to hypoxia<br>• peptidyl-proline hydroxylation to 4-hydroxy-L-proline	• regulation of oxidative stress-induced intrinsic apoptotic signaling pathway<br>• response to ER stress
**ERO1A;** ER oxidoreductase 1 alpha (A5PJN2)	0.74	3,44E-02	ER membrane	• 4-hydroxyproline metabolic process<br>• cell redox homeostasis<br>• cellular protein modification<br>• chaperone cofactor-dependent protein refolding	• intrinsic apoptotic signaling pathway in response to ER stress<br>• ER unfolded protein response<br>• protein folding in ER<br>• protein maturation by protein folding<br>• release of sequestered calcium ion into cytosol
**HSPB1;** heat shock protein family B (small) member 1 (E1BEL7)	0.74	1,72E-02	Nucleus and Plasma membrane	• chaperone-mediated protein folding<br>• intracellular signal transduction<br>• negative regulation of oxidative stress-induced intrinsic apoptotic signaling pathway	• positive regulation of interleukin-1 beta production<br>• positive regulation of tumor necrosis factor biosynthetic process<br>• regulation of I-kappaB kinase/NF-kappaB signaling

**MANF**; mesencephalic astrocyte derived neurotrophic factor (P80513)	0.74	1,49E-04	Extracellular Space	• response to unfolded protein	•
**SEC62;** SEC62 homolog, preprotein translocation factor (Q32PD3)	0.74	9,02E-03	Rough ER	• protein transporter activity	
**LAP3;** leucine aminopeptidase 3 (G3N0I4)	0.75	2,18E-02	Cytoplasm	• aminopeptidase activity<br>• manganese ion binding	• metalloexopeptidase activity
**ACO2;** Aconitate hydratase, mitochondrial (Q1P9Q3)	0.75	1,30E-04	Mit.	• tricarboxylic acid cycle	
**HSPA9;** heat shock protein family A (Hsp70) member 9 (Q3ZCH0)	0.76	1,63E-07	Nucleolus and Mit.	• unfolded protein binding<br>• protein folding	• iron-sulfur cluster assembly
**PDIA4;** protein disulfide isomerase family A member 4 (F1MEN8)	0.76	2,04E-03	ER lumen	• cell redox homeostasis	
**OAT;** ornithine aminotransferase (F1MYG0)	0.77	4,42E-04	Cytoplasm	• protein hexamerization	
**HSPE1;** heat shock protein family E (Hsp10) member 1 (P61603)	0.77	4,99E-02	Mit. matrix	• chaperone cofactor-dependent protein refolding	• response to unfolded protein
**HNRNPC;** heterogeneous nuclear ribonucleoprotein C (C1/C2) (Q3SX47)	0.77	1,73E-02	nucleus	• mRNA binding	
**CANX;** calnexin (A7Z066)	0.77	8,53E-08	ER	• clathrin-dependent endocytosis<br>• protein folding	• calcium ion binding<br>• unfolded protein binding
**HSP90B1;** heat shock protein 90 beta family member 1 (Q95M18)	0.78	7,02E-03	ER lumen	• negative regulation of apoptosis<br>• protein folding<br>• regulation of phosphoprotein	• response to hypoxia<br>• retrograde protein transport, ER to cytosol<br>• ubiquitin-dependent ERAD pathway
**SSR1;** signal sequence receptor subunit 1, Translocon-associated protein subunit alpha (A6QLP7)	0.79	4,34E-02	ER membrane	• cotranslational protein targeting to membrane	
**LRPAP1;** LDL receptor related protein associated protein 1 (F6RBT3)	0.79	7,04E-03	rough ER lumen, Endosome, Golgi, PM	• low-density lipoprotein particle receptor binding<br>• negative regulation of very-low-density lipoprotein particle clearance<br>• negative regulation of cell death	• negative regulation of protein binding<br>• negative regulation of receptor internalization

**PRKCSH;** protein kinase C substrate 80K-H (Q28034)	0.79	1,31E-04	ER	• calcium ion binding	• in utero embryonic development<br>N-glycan processing
**ASPH;** aspartate beta-hydroxylase (Q28056)	0.80	2,18E-02	ER membrane	• activation of cysteine-type endopeptidase activity<br>• peptidyl-aspartic acid hydroxylation	• regulation of protein depolymerization<br>• regulation of protein stability<br>• negative regulation of cell proliferation
**CKAP4;** cytoskeleton associated protein 4 (F1ME65)	0.80	4,88E-05	ER, nucleus cytoplasm,	• Post-translational protein phosphorylation.	
**LMNB2;** lamin B2 (F1MJI7)	0.81	1,31E-04	Cytoskeleton, and nucleus	• structural molecule activity	
**ERP29;** ER protein 29 (P81623)	0.81	3,88E-02	ER lumen	• activation of MAPK activity<br>• intracellular protein transport<br>• negative regulation of gene expression<br>• negative regulation of protein secretion	• protein folding<br>• protein secretion<br>• regulation of ER stress-induced intrinsic apoptotic signaling pathway
**LMNA**; lamin A/C (F1MYG5)	0.81	4,03E-02	Cytoskeleton, and nucleus	• cellular response to hypoxia<br>• establishment or maintenance of microtubule cytoskeleton polarity<br>• response to stress<br>• negative regulation of extrinsic apoptotic signaling pathway	• negative regulation of release of cytochrome c from mitochondria<br>• nuclear envelope organization<br>• regulation of protein localization to nucleus<br>• sterol regulatory element binding protein import into nucleus
**VDAC2;** voltage dependent anion channel 2 (P68002)	0.81	5,28E-03	MOM	• binding of sperm to zona pellucida<br>• negative regulation of intrinsic apoptotic signaling pathway	• negative regulation of protein polymerization
**ATP5B;** ATP synthase, H+ transporting, mitochondrial F1 complex, β polypeptide (P00829)	0.83	1,16E-02	MIM	• ATP synthesis coupled proton transport	• lipid metabolic process
**SERPINH1;** serpin family H member 1 (Q2KJH6)	0.83	3,28E-02	ER lumen	• collagen biosynthetic process<br>• protein maturation	
**RPN1;** ribophorin I (A3KN04)	0.84	1,79E-02	ER membrane	• protein N-linked glycosylation via asparagine	

**HADHA;** hydroxyacyl-CoA dehydrogenase trifunctional multienzyme complex subunit alpha (Q3SZ00)	0.84	2,14E-02	MIM	• fatty acid beta-oxidation	• response to insulin
**DHX9;** DExH-box helicase 9 (G5E5N3)	0.85	4,99E-02	Cytoskeleton, nucleus	• alternative mRNA splicing, via spliceosome<br>• cellular response to tumor necrosis factor<br>• positive regulation of cytoplasmic translation<br>• positive regulation of DNA repair	• positive regulation of NF-kappaB transcription factor activity<br>positive regulation of response to cytokine stimulus<br>• positive regulation of RNA export from nucleus<br>• regulation of mRNA processing<br>RNA secondary structure unwinding
**ATP5A1;** ATP synthase, H+ transporting, mitochondrial F1 complex, α subunit 1, (F1MLB8)	0.85	1,93E-02	Mit.	• ATP synthesis coupled proton transport<br>lipid metabolic process	
**IMMT;** inner membrane mito-chondrial protein, MICOS complex subunit MIC60 (F1MXX0)	0.87	4,99E-02	MIM	• cristae formation	• mitochondrial calcium ion homeostasis
**ITGB1;** integrin subunit beta 1 (P53712)	0.87	4,35E-02	Endosome, Plasma membrane	• cell adhesion mediated by integrin<br>• cell-matrix adhesion<br>• cellular response to low-density lipoprotein particle stimulus<br>• integrin-mediated signaling pathway	• positive regulation of protein localization to plasma membrane<br>receptor internalization<br>• regulation of collagen catabolic process
**PHB;** prohibitin (Q3T165)	0.89	4,99E-02	MIM	• DNA biosynthetic process	• negative regulation of transcription from RNA polymerase II promoter
**HNRNPM;** heterogeneous nuclear ribonucleoprotein M (F1MY44)	0.89	4,03E-02	Nucleus	• mRNA splicing, via spliceosome	

Supplementary Table 1c. Proteins with increased abundance due to PA exposure in oocytes and their GO biological processes.

Symbol; Entrez Gene Name (GenPept/UniProt/Swiss-Prot Accession)	**FC**	**Expr p-value**	**Cubcellular location**	**GO Biological processes**	
CYCS; cytochrome c, somatic (P62894)	2.382	3,03E-02	Mit. intermembrane space. Associated with MIM.	• mitochondrial electron transport, cytochrome c to oxygen<br>• mitochondrial electron transport, ubiquinol to cytochrome c	• apoptotic process<br>• positive regulation of cysteine-type endopeptidase activity involved in apoptotic process
PSAP; prosaposin (A1L555)	1.977	5,61E-05	lysosome	• sphingolipid metabolic process	•
SLC25A5; solute carrier family 25 member 5, ADP/ATP translocase 2 (G3N3W3)	1.828	1,69E-03	MIM	• negative regulation of mitochondrial outer membrane permeabilization involved in apoptotic signaling pathway	• positive regulation of cell proliferation
COPS3; COP9 signalosome subunit 3 (A6H7B5)	1.824	3,03E-02	Nucleus and cytoplasm	• in utero embryonic development<br>• protein deneddylation	• ubiquitin-dependent protein catabolic process
GSR; glutathione-disulfide reductase (E1BKZ1)	1.786	3,03E-02	Mit.	• cell redox homeostasis	• glutathione metabolic process
MYDGF; myeloid derived growth factor (P62248)	1.741807	3,28E-03	Extracellular Space and Endoplasmic reticulum-Golgi intermediate compartment	• positive regulation of<br>○ MAPK cascade<br>○ Pi3K signaling<br>○ protein kinase B signaling<br>• negative regulation of apoptosis	○ protein phosphorylation<br>○ transcription from RNA polymerase II promoter
UQCRB; ubiquinol-cytochrome c reductase binding protein (P00129)	1.713957	1,57E-03	MIM	• mitochondrial electron transport, ubiquinol to cytochrome c<br>• mitochondrial respiratory chain complex III assembly	• aerobic respiration
RAB3D; RAB3D, member RAS oncogene family (E1BNX1)	1.698244	3,39E-02	Mit. , cytoplasmic microtubule	• peptidyl-cysteine methylation	• positive regulation of regulated secretory pathway
ZC2HC1A; zinc finger C2HC-type containing 1A (G1K1W5)	1.694338	4,53E-03		•	•

BCAP31; B-cell receptor associated protein 31 (Q5E9F1)	1.648162	2,22E-04		• calcium-mediated signaling using intracellular calcium source<br>• ER to Golgi vesicle-mediated transport<br>• intracellular protein transport<br>• negative regulation of endoplasmic reticulum calcium ion concentration<br>• protein localization to endoplasmic reticulum exit site	• positive regulation of<br>o cysteine-type endopeptidase activity involved in apoptotic process<br>o cytosolic calcium ion concentration<br>o ER-associated ubiquitin-dependent protein catabolic process<br>o intrinsic apoptotic signaling pathway<br>o mitochondrial Ca ion concentration<br>o retrograde protein transport, ER to cytosol
RAB2B; RAB2B, member RAS oncogene family (E1BC58)	1.625549	3,03E-02	PM, Golgi membrane	• positive regulation of exocytosis	
GDI2; GDP dissociation inhibitor 2 (P50397)	1.563148	1,28E-04	Cytoplasm, and PM	• small GTPase mediated signal transduction	• protein transport<br>•
MAP2K2; mitogen-activated protein kinase kinase 2 (Q17QH2)	1.534617	3,23E-02	Cytoplasm	• activation of protein kinase activity ERK1 and ERK2 cascade<br>• regulation of apoptotic process<br>• regulation of mitotic cell cycle	• signal transduction by protein phosphorylation<br>• stress-activated protein kinase signaling cascade
NASP; nuclear autoantigenic sperm protein (Q2T9P4)	1.524053	5,71E-03	Nucleus and cytoplasm	• histone binding<br>• Hsp90 protein binding<br>• blastocyst development<br>• histone exchange<br>• protein transport	• DNA replication<br>• DNA replication-dependent nucleosome assembly<br>• DNA replication-independent nucleosome assembly
PCBD1; pterin-4 alpha-carbinolamine dehydratase 1 (Q3ZBD3)	1.520548	3,61E-02	Nucleus and cytoplasm	• protein heterooligomerization<br>• transcription, DNA-templated	• protein homotetramerization regulation of transcription, DNA-templated

HSPA8; heat shock protein family A (Hsp70) member 8 (A8KC76)	1.520548	1,69E-03	Nucleus and cytoplasm	• ATP metabolic process<br>• chaperone cofactor-dependent protein refolding<br>• chaperone-mediated autophagy<br>• chaperone-mediated autophagy translocation complex disassembly<br>• chaperone-mediated protein transport involved in chaperone-mediated autophagy<br>• late endosomal microautophagy<br>• membrane organization<br>• regulation of cell cycle<br>• response to unfolded protein<br>• clathrin coat disassembly	• cytokine-mediated signaling pathway<br>• protein folding, methylation and refolding<br>• protein targeting to lysosome involved in chaperone-mediated autophagy<br>• regulation of mRNA stability<br>• mRNA splicing, via spliceosome<br>• positive regulation of mRNA splicing, via spliceosome<br>• negative regulation of transcription, DNA-templated<br>• regulation of protein complex assembly and stability<br>• regulation of protein import and stability
NUP35; nucleoporin 35 (A5PJI3)	1.513561	4,16E-02	Nucleus	• nucleic acid binding<br>• mRNA transport	• nucleocytoplasmic transport<br>• protein transport
QDPR; quinoid dihydropteridine reductase (Q3T0Z7)	1.496236	1,69E-02	Mit.	• tetrahydrobiopterin biosynthetic process	
RAB7A; RAB7A, member RAS oncogene family (F1MJQ1)	1.482518	3,81E-02	late endosome membrane, Golgi, Lysosome;<br>and lipid droplet	• lipophagy	
P4HB; prolyl 4-hydroxylase subunit beta, Protein disulfide-isomerase (A6H7J6)	1.475707	5,61E-05	ER chaperone complex, and ER-Golgi intermediate compartment	• regulation of oxidative stress-induced intrinsic apoptotic signaling pathway response to endoplasmic reticulum stress	• cell redox homeostasis cellular response to hypoxia
RPL27A; ribosomal protein L27a (Q28150)	1.448772	3,03E-02	cytosolic large ribosomal subunit, ER	• cytoplasmic translation<br>• nuclear-transcribed mRNA catabolic process,	
**AKR1B1;** aldo-keto reductase family 1 member B (Q5E962)	1.438799	4,16E-02	nucleus and cytoplasm	• oxidoreductase activity	• negative regulation of apoptotic process

**CTTN;** cortactin (Q1RMR3)	1.432188	5,71E-03	cortical cytoskeleton, mitotic spindle midzone, Golgi apparatus	• negative regulation of extrinsic apoptotic signaling pathway<br>• regulation of autophagy of mitochondrion	• positive regulation of actin filament polymerization<br>• actin filament polymerization<br>• intracellular protein transport
**HADHB;** hydroxyacyl-CoA dehydrogenase trifunctional multienzyme complex subunit beta, Mitochondrial trifunctional protein, beta subunit (A5D9E7)	1.406048	3,02E-02	ER, Mit., MIM, MOM	• cardiolipin acyl-chain remodeling<br>• fatty acid beta-oxidation	
**PEBP1;** phosphatidyl-ethanolamine binding protein 1 (P13696)	1.399587	4,16E-02	Cytoplasm	• MAPK cascade	
**PDIA3;** protein disulfide isomerase family A member 3 (A5D7E8)	1.367729	2,59E-02	endoplasmic reticulum	• cell redox homeostasis<br>• positive regulation of extrinsic apoptotic signaling pathway	
**CCT8;** chaperonin containing TCP1 subunit 8 (Q3ZCI9)	1.358313	3,81E-02	Cytoskeleton	• de novo' protein folding<br>• chaperone-mediated protein folding	
**PRDX3;** peroxiredoxin 3 (Q3MHF7)	1.352073	4,93E-02	Mit.<br>Mit. matrix<br>Endosome<br>Early endosome	• cell redox homeostasis<br>• cellular response to reactive oxygen species<br>• response to hydrogen peroxide<br>• hydrogen peroxide catabolic process<br>• mitochondrion organization<br>• regulation of mitochondrial membrane potential	• negative regulation of kinase activity<br>• peptidyl-cysteine oxidation<br>• positive regulation of NF-kappaB transcription factor activity<br>• positive regulation of cell proliferation<br>• response to lipopolysaccharide
**PABPC4;** poly(A) binding protein cytoplasmic 4 (A4IFC3)	1.336596	3,03E-02	Cytoplasm	• mRNA binding	• poly(A) binding and regulation of mRNA stability
**ELOB;** elongin B (Q3SZ32)	1.336596	3,03E-02	Nucleus	• translation elongation factor activity<br>• ubiquitin protein ligase binding<br>• post-translational protein modification<br>• protein complex assembly<br>• protein ubiquitination	• regulation of transcription from RNA polymerase II promoter in response to hypoxia<br>• transcription elongation from RNA polymerase II promoter<br>• transcription from RNA polymerase II promoter

**YWHAG;** tyrosine 3-monooxygenase/tryptophan 5-monooxygenase activation protein gamma (A7Z057)	1.297179	3,03E-02	Cytoplasm	• positive regulation of protein insertion into mitochondrial membrane involved in apoptotic signaling pathway<br>• regulation of G2/M transition of mitotic cell cycle<br>• G2/M transition of mitotic cell cycle<br>• membrane organization	• cellular response to insulin stimulus<br>• protein targeting<br>• negative regulation of protein kinase activity<br>• promotes cell survival
**FKBP4;** FK506 binding protein 4 (F1MU79)	1.279381	4,16E-02	nucleoplasm and cytoplasm	• chaperone-mediated protein folding<br>• protein complex localization<br>• steroid hormone receptor complex assembly	• embryo implantation<br>• male sex differentiation
**STIP1;** stress induced phosphoprotein 1 (Q3ZBZ8)	1.25603	3,39E-02	Nucleus	• Acts as a co-chaperone for HSP90AA1.<br>• Mediates the association of the molecular chaperones HSPA8/HSC70 and HSP90.	• Hsp70 protein binding<br>• response to stress
**PPA1**; pyrophosphatase (inorganic) 1 (P37980)	1.2218	3,03E-02	Cytoplasm	• phosphate-containing compound metabolic process	
**NLRP5;** NLR family pyrin domain containing 5 (F1MT67)	1.164126	2,59E-02	Cytoplasm and germinal vesicle	• As a member of the subcortical maternal complex (SCMC), plays an essential role for zygotes to progress beyond the first embryonic cell divisions.	

Supplementary Table 1d. Proteins with decreased abundance due to PA exposure in oocytes and their GO biological processes.

Symbol; Entrez Gene Name (UniProt Accession)	FC	Expr p-value	Cubcellular location	• GO Biological processes	
**VIM;** vimentin (P48616)	0.230	5,16E-04	nucleus, ER, and Mit.,	• positive regulation of translation<br>• regulation of mRNA stability	• SMAD protein signal transduction
**ALB;** albumin (B0JYQ0)	0.257	5,61E-05	Extracellular Space		
**ARL8B;** ADP ribosylation factor like GTPase 8B (F2Z4I5)	0.520	2,05E-02	spindle midzone and lysosomal membrane	• chromosome segregation<br>• lysosome localization	• small GTPase mediated signal transduction
**ZP3;** zona pellucida glycoprotein 3 (A6H6X7)	0.520	3,16E-02	Extracellular Space	• binding of sperm to zona pellucida<br>• blastocyst formation<br>• oocyte development<br>• phosphatidylinositol-mediated signaling	• positive regulation of acrosome reaction<br>• positive regulation of calcium ion import<br>• positive regulation of transcription, DNA-templated
**TMEM43;** transmembrane protein 43 (A6QQR5)	0.582	3,03E-02	ER lumen, nuclear inner membrane	• nuclear membrane organization	
**RPS6;** ribosomal protein S6 (F1MKZ5)	0.601	3,23E-02	Cytoplasm	• translation	
**HINT2;** histidine triad nucleotide binding protein 2 (Q8SQ21)	0.613762	3,03E-02	Mit.	• apoptotic process<br>• multicellular organismal lipid catabolic process	• negative regulation of peptidyl-lysine acetylation<br>• steroid biosynthetic process
**HSPA5**; heat shock protein family A (Hsp70) member 5 (Q0VCX2)	0.656145	2,99E-02	ER lumen	• maintenance of protein localization in endoplasmic reticulum	
**GCAT;** 2-amino-3-ketobutyrate coenzyme A ligase, mitochondrial (Q0P5L8)	0.660693	3,03E-02	Mit. and Nucleus	• biosynthetic process L-threonine catabolic process to glycine	
**ENO1;** enolase 1 (F1MB08)	0.669885	5,38E-03	Cytoplasm and plasma membrane	• negative regulation of hypoxia-induced intrinsic apoptotic signaling pathway	• positive regulation of ATP biosynthetic process
**POR;** cytochrome p450 oxidoreductase (A5D9D3)	0.677642	3,03E-02	ER membrane	• cellular organofluorine metabolic process	• positive regulation of monooxygenase activity

**ASAH1;** N-acylsphingosine amidohydrolase 1, Acid ceramidase (Q17QB3)	0.695024	4,16E-02	Lysosome	• ceramidase activity	• lipid metabolic process
**TMED10;** transmembrane p24 trafficking protein 10 (Q5E971)	0.711214	4,16E-02	ER membrane, Golgi , and PM	• COPI-coated vesicle budding protein transport<br>• regulation of amyloid-beta formation	• retrograde vesicle-mediated transport, Golgi to ER
**GNS;** glucosamine (N-acetyl)-6-sulfatase (F1MXZ0)	0.724436	1,08E-02	Lysosome	• glycosaminoglycan metabolic process	
**NLRP13;** NLR family pyrin domain containing 13 (G3MYV6)	0.731139	1,69E-03		• inflammation	
**ALDH1A1;** aldehyde dehydrogenase 1 family member A1 (P48644)	0.753356	3,02E-02	Cytoplasm	• oxidation-reduction process retinoid metabolic process	
**ACAT1;** Acetyl-CoA acetyltransferase, mitochondrial (Q29RZ0)	0.755092	3,23E-02	Mit.	• acetyl-CoA biosynthetic process<br>• acetyl-CoA catabolic process<br>• coenzyme A biosynthetic process<br>• fatty acid beta-oxidation	• isoleucine catabolic process<br>• ketone body catabolic process<br>• propionyl-CoA biosynthetic process
**PTGFRN;** prostaglandin F2 receptor inhibitor (F1MT41)	0.778037	3,03E-02	PM	• lipid particle organization	
**CCT7;** chaperonin containing TCP1 subunit 7, T-complex protein 1 subunit eta (F1MWR8)	0.778037	3,03E-02		• binding of sperm to zona pellucida positive regulation of establishment of protein localization to telomere<br>• positive regulation of protein localization to Cajal body	• positive regulation of telomerase RNA localization to Cajal body<br>• positive regulation of telomere maintenance via telomerase protein folding<br>• protein stabilization<br>• toxin transport
